# The role of mass media exposure on tuberculosis knowledge and attitude among migrant and seasonal farmworkers in Northwest Ethiopia

**DOI:** 10.1186/s12879-020-05316-9

**Published:** 2020-08-05

**Authors:** Kassahun Alemu Gelaye, Getu Debalkie, Tadesse Awoke Ayele, Sintayehu Daba Wami, Malede Mequanent Sisay, Destaw Fetene, Haileab Fekadu Wolde, Temesgen Yihunie Akalu

**Affiliations:** 1 Department of Epidemiology and Biostatistics, Institute of Public Health, University of Gondar, Gondar, Ethiopia; 2 Department of Health Education and Behavioral Science, Institute of Public Health, University of Gondar, Gondar, Ethiopia; 3 Department of Environmental and Occupational Health and Safety, Institute of Public Health, University of Gondar, Gondar, Ethiopia

**Keywords:** Knowledge, Attitude, TB, Migrants, And seasonal farmworkers, West Gondar

## Abstract

**Background:**

Globally, tuberculosis (TB) is the 10th leading cause of death. Despite no country achieved its target, the world health organization (WHO) proposed a 90–90-90 approach to fastening the end TB strategy. Improvement and progression of TB control need good knowledge and a favorable attitude towards the disease. However, interventions designed don’t take migrants and seasonal farmworkers into account. Therefore, this study aimed at estimating the level of knowledge and attitude on Tuberculosis among migrant and seasonal farmworkers in northwest Ethiopia.

**Methods:**

Community-based cross-sectional study was conducted in the West Gondar zone from October to November 2018. A two-stage cluster sampling was used to select 949 migrant and seasonal farmworkers. Both bivariate and multivariable logistic regression analyses were performed. A *p*-value of < 0.05 was used to declare statistical significance. The goodness of fit was checked using Hosmer and Lemeshow test.

**Results:**

In this study, (41.8%), (95% CI: 38.73, 45.01) and (50.5%), (95% CI: 47.29, 53.65) of migrants and seasonal farmworkers had good knowledge and a favorable attitude, respectively. The odds of good knowledge among mass media exposed migrants were AOR = 1.42, 95% CI: (1.02, 2.01). Moreover, urban residence and having good knowledge increase the odds of favorable attitude by 1.66, (AOR = 1.7; 95% CI: 1.05, 2.62) and 4.3 (AOR = 4.3, 95%CI: 3.26, 5.75), respectively.

**Conclusion:**

In this study, the overall knowledge and attitude of migrant and seasonal farmworkers on TB were low. Family size and mass media exposure significantly affect knowledge of the migrants on TB. On the other hand, the attitude was affected by urban residence, health information, and having good knowledge. Health promotion interventions, focused on TB cause, mode of transmission, prevention, and treatment are important to migrant and seasonal farmworkers to improve the knowledge and attitude of migrants and seasonal farmworkers.

## Background

Tuberculosis (TB) is caused by mycobacterium species which is treatable and curable and it mainly affects the lungs [1]. According to the World Health Organization (WHO) 2019 report, TB resulted in 1.2 million deaths and stands as the 10th leading cause of death, ranking above HIV/AIDS. Ethiopia is among the 30 TB high burden and 27 high Multi-Drug Resistance (MDR) TB burden countries globally. There was a 27% reduction from 1.7 million in 2000 and a 60% reduction from 620,000 in 2000 among HIV negative and HIV positive individuals, respectively [1]. However, according to a global plan to end-TB between 2016 and 2020 no country across the globe achieved a 90–90-90 target [2]. A 90–90-90 global target is defined as by the year 2020 90% of all people living with HIV will know their HIV status, 90% of all the people diagnosed with HIV infection will receive sustained antiretroviral therapy, and 90% of all people receiving antiretroviral therapy will have viral suppression.

A systematic review and meta-analysis showed that the prevalence of MDR-TB in Ethiopia was 1.4% [3]. Ethiopia has implemented several TB control efforts including TB program capacity strengthening at the central and regional level, the involvement of private sectors in TB diagnosis and treatment, expansion of culture, and introduction of Gene-expert for diagnosis, and expansion of MDR treatment centers. As a result, the mortality rate declined from 73 to 32%, treatment success rate improved from 79 to 90%, and the case detection rate increase from 33 to 62% in the year 2005 to 2014 [4].

Further improvements and progression of TB control need a good understanding of the cause, mode of transmission, prevention and treatment, and favorable attitude. Addressing knowledge gaps in TB prevention has a great role in eliminating TB [5]. Literacy [6, 7], gender [6], mass media [8–10], is a TB patient [11], professional occupation [7, 12], health education [13], culture myths [14, 15], knowledge [16], wealth index [17], age [18], and residence [19] were some of the factors affecting knowledge and attitude. Since knowledge is a precursor of a TB control strategy, determining knowledge of migrants and seasonal farmworkers is very crucial in enabling and fastening controlling TB strategies [20].

Despite significant improvement in TB control and prevention in the country, the evidence on the current knowledge, attitude, and associated factors towards TB in Ethiopia among migrant and seasonal farmworkers are limited. Hence, estimating their knowledge and attitude has a vital role in undertaking measures to fasten the motto of the end TB strategy.

Migrants and seasonal farmworkers are highly vulnerable to Tuberculosis since they are disadvantaged groups. However, to fasten TB control strategy *emphasis is only given to smear-positive patients and contact tracing is implemented strongly to minimize the burden of TB* [21]. *Therefore, a*ddressing the risk of TB in international migrants is an essential component of TB prevention and care efforts in developing countries, and strategies to systematically screen for, diagnose, treat and prevent TB among this group contribute to national and global TB elimination goals. It is, therefore, this study was aimed at estimating knowledge, attitude, and associated factors on migrant and seasonal farmworkers on TB in the West Gondar zone, northwest Ethiopia.

## Methods

### Study setting

**Fig. 1:**
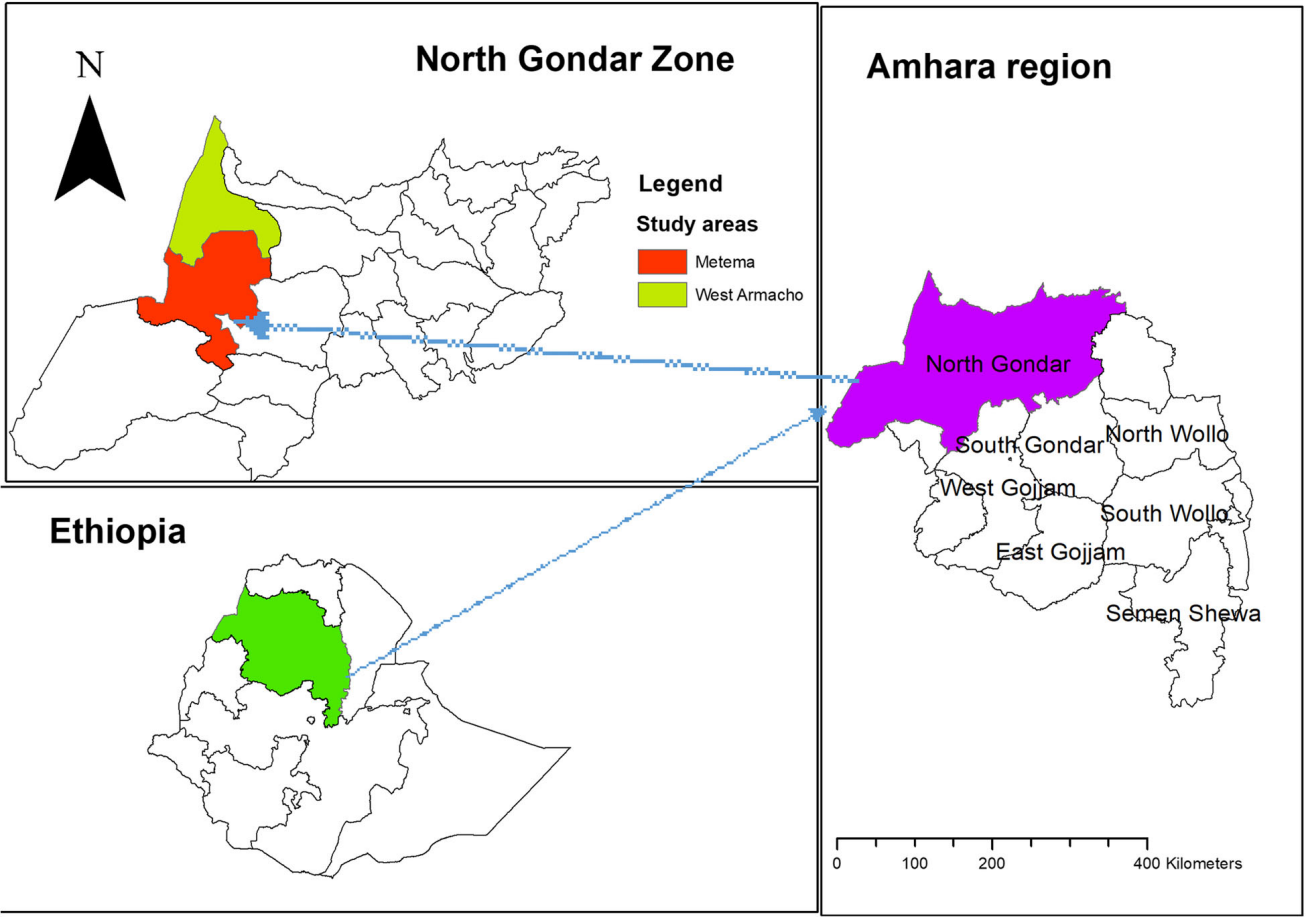
Map of the study area (constructed by authors using ArcGIS version 10.6)

The study was implemented in Amhara Regional State, northwest Ethiopia. Among 167 districts found in the region, migrants and seasonal farmworkers went for work mainly in Metema and West Armachiwo districts. These districts are the two common sites of sesame production in the region where hundreds of thousands of migrants and seasonal farmworkers traveled during planting, weeding, and harvesting seasons **(**Fig. 1**)**.

### Population, sample size determination, and procedure

All seasonal and migrant farmworkers in the West Gondar zone from October 2018 to November 2018 were included in the study. A pilot study was conducted among 50 migrants and seasonal farmworkers in Quara district, West Gondar zone to determine the minimum sample size. Then sample size was determined using a single population formula by using the following assumptions. Based on the evidence from the pilot study the proportion of good knowledge and favorable attitude were 44 and 53%, respectively. Besides, 95% CI, 5% margin of error and a 10% non-response rate were used. Since the sampling technique was cluster; the design effect of 2 was also used. Therefore, a total of 796 and 804 study participants were required for knowledge and attitude, respectively. To estimate predictors, the sample size was computed using assumptions of power, 80, 95%CI, odds ratio 1.5–1.8 for the predictors of health information, occupation, and knowledge. Finally, the largest sample size of 976 was used.

A two-stage cluster sampling technique was used. In the first stage, the farm companies were randomly selected, and then seasonal migrant workers were randomly selected.

### Data collection tool and procedure

A pretested and structured questionnaire was used to collect data on knowledge, attitude, and health-seeking behavior on TB. The tool was prepared first in English then translated into the local language (Amharic). Then it was re-translated to English by language experts to keep its consistency. The questionnaire consisted of 43 questions, divided into two parts. The first part addressed socio-demographic characteristics and sources of information. The second component includes TB knowledge, attitude, and health-seeking behavior. The data was collected by interviewing through 10 trained BSc nurses. Four supervisors control the overall data collection process. We had two groups of data collectors. The first group which consisted of 5 data collectors enrolled in the Metema district and other equal numbers of data collectors collect the data in the West Armacho district. Before going to actual data collection, two-day training was given to all data collectors and supervisors. Supervisors closely monitored the interviewing process on a daily base.

In this study, the outcome variables were knowledge about TB and attitude about TB. Briefly, knowledge was assessed by 23 questions that addressed the etiology, symptoms, transmission, prevention, and treatment. Respondents who answered 12 (> = 50%) of these questions were defined as having good knowledge and vice versa. Furthermore, the attitude was assessed by five-item Likert scale questions and respondents who had a positive attitude towards three of the questions (60%) were classified as having a favorable attitude. The independent variables include: region, sex, age, family size, education status, occupation, residence, religion, marital status, income, number of visits, and length of stay were included. The income status of respondents was dichotomized into low and high income. Low income was defined as respondents who gain less than the median (120 birr’s). Moreover, the source of information data was also collected from respondents.

### Statistical analysis

Epi Data version 3.1 was used for data entry and the analysis was performed using Stata version 14. Coding, recoding, computing, and data cleaning were computed. Frequencies and proportions were used to describe the data. A chi-square test was checked to assess the relationship between two categorical variables. The bivariate analysis was performed and variables that were significant at *p*-value < 0.2 were entered in the multivariable analysis. To examine the association between independent variables and the outcome variables multivariable logistic regression analysis was performed. In the multivariable logistic regression analysis, *p-*value < 0.05 was used to declare statistical significance association. Multi-collinearity was assessed using the variance inflation factor (VIF) and considered no multi-collinearity lower than 10. Odds ratio with 95%CI was used to measuring the strength of the association. The goodness of fit was 0.54 and 0.76 for knowledge and attitude, respectively.

## Results

### Socio-demographic and economic factors

The response rate of the study was 97.23%. The mean age of respondents was 26.05 ± 7.82 years. Of all respondents, 917 (96.6%) were from the Amhara region. The majority, 728 (76.21%) were in the age group of 13–29 years. The median family size of respondents was 5 with the Inter Quartile Range (IQR = 4–6). Of all, 447 (47.1%) had no formal education. About 644 (68%) and177 (19%) of migrants and seasonal workers were farmers and students by occupation, respectively. The median daily income of respondents was 120 Birr with IQR of 100–150 Birr. Regarding residence, 831 (87.57%) were rural dwellers. Only 178 (18.76%) of migrant and seasonal farmworkers had one visit (Table 1).

**Table 1:** Socio-demographic and economic characteristics of migrant and seasonal farmworkers in West Gondar zone, northwest Ethiopia 2018 (*N* = 949)

Variables	Frequency (%)
**Region**	
Tigray	27 (2.8)
Amhara	917(96.6)
Oromia	5 (0.5)
**Sex**	
Male	942 (99.3)
Female	7(0.7)
**Age**	
13–20	244 (25.7)
21–29	484 (51.0)
30–39	151 (15.9)
40–67	70 (7.4)
**Family size in their family**	
1–2	88 (9.3)
3–5	536 (56.5)
6–14	325 (34.2)
**Educational status**	
Unable to read and write	374 (39.4)
Able to read and write	73 (7.7)
Primary education	405 (42.7)
Secondary education	97 (10.2)
**Occupation before departure**	
Student	177 (18.7)
Farmer	644 (67.8)
Unemployed	128 (13.5)
**Residence**	
Rural	831 (87.6)
Urban	118 (12.4)
**Religion**	
Orthodox	931 (98.1)
Muslim	15 (1.6)
Protestant	1 (0.1)
Catholic	2 (0.2)
**Marital status**	
Single	648 (68.3)
Married	243 (25.6)
Divorce	58 (6.1)
**Income**	
Low	496 (52.3)
High	453 (47.7)
**Number of visits to the farms**	
First	178 (18.8)
2–4	293 (30.9)
5–8	280 (29.5)
≥ 9	198 (20.8)
**Length of stay**	
Less than two month	576 (60.7)
Two months and above	373 (39.3)

### Source of information of respondents

Five hundred sixty-five (59.54%) heard information about TB at different sources. Of these, 328 (58.05%) heard from mass media, and the majority, 508 (90.07%) obtained health information from health workers. Also, 270 (47.96%) obtained from friends/families, 90 (15.96%) reading posters and 145 (25.75%) from school.

#### Knowledge on tuberculosis

In this study, 758 (79.87%) of respondents be acquainted with the cause of TB as germ/bacteria. Regarding symptoms of TB, 721(75.97%) respondents and nearly half (45.63%) know TB patients had persistent cough and sputum with blood, respectively. Nearly one-third of 351 (36.99%) of respondents know that TB patients had a fever. Of all, 732 (77.13%) of respondents know that TB is transmittable. Five hundred thirty-three, 533 (56.16%) know that TB transmission can be prevented by minimizing close contact. Regarding on treatment of TB, 785 (82.72%) know that TB can be prevented. In this study, 41.83% (95%CI: 38.73, 45.01) of migrant and seasonal farmworkers were knowledgeable (Table 2).

**Table 2:** Knowledge on Cause, symptoms, methods of transmission and prevention of tuberculosis among migrant and seasonal farmworkers in West Gondar zone, 2018 (*n* = 949)

Variables	Frequency (%)
**Cause of Tuberculosis germ/bacteria**	
Yes	758(79.9)
No	191 (20.1)
**Persistent cough**	
Yes	721(75.97)
No	228(24.03)
**Sputum with blood**	
Yes	433 (45.63)
No	516 (54.37)
**Fever**	
Yes	351 (37.0)
No	598 (63.0)
**Poor apatite**	
Yes	381 (40.2)
No	568 (59.8)
**Night sweating**	
Yes	318 (33.5)
No	631 (66.5)
**Weight loss**	
Yes	412 (43.4)
No	537 (56.6)
**Chest pain**	
Yes	496 (41.7)
No	553 (58.3)
**Transmit through cough/sneezing**	
Yes	658 (69.3)
No	291 (30.7)
**Via touching TB person**	
Yes	219 (23.1)
No	730 (76.9)
**Through sharing utensils**	
Yes	521 (54.9)
No	428 (45.1)
**By sexual contact**	
Yes	168 (17.7)
No	781 (82.3)
**By drink unboiled milk**	
Yes	240 (25.3)
No	709 (74.7)
**TB is preventable**	
Yes	686 (72.4)
No	262 (27.6)
**Minimizing close contact**	
Yes	533 (56.2)
No	416 (43.8)
**Covering mouth while coughing**	
Yes	468 (49.3)
No	481 (50.7)
**Avoid sharing utensils**	
Yes	445 (46.9)
No	504 (53.1)
**Early treatment**	
Yes	410 (43.2)
No	539 (56.8)
**Good nutrition**	
Yes	315 (33.2)
No	634 (66.8)
**Using separate rooms**	
Yes	371 (39.1)
No	578 (60.9)
**Close opening windows**	
Yes	232 (24.4)
No	717 (75.6)
**Using modern drugs**	
Yes	762 (78.3)
No	211 (21.7)
**Nutritional support**	
Yes	38 (3.9)
No	935 (96.1)
**TB is not treatable**	
Yes	192 (20.2)
No	757 (79.8)
**Overall TB knowledge**	
Poor knowledge	552 (58.2)
Good knowledge	397 (41.8)

#### The attitude of respondents on tuberculosis

The majority, 655 (67.93%) believed TB is a very serious disease and only 294 (30.2%) believed TB is a serious problem in their community. Six hundred eighty-three (72.0%) mentioned that they would not feel feared or ashamed if they would have TB. Of all, 275 (28.35%) believed that some people are more likely to be affected by TB than others. Three hundred sixty-six (38.57%) would not show any feeling of compassion and desire to help TB patients. The overall favorable attitude was observed in nearly 50 % of respondents 50.47% (95% CI: 47.29–53.65) **(**Table 3**)**.

**Table 3:** Attitude of respondents on Tuberculosis among migrant and seasonal workers in West Gondar, 2018 (*N* = 949)

Variables	Frequency (%)
**How serious are the diseases TB?**	
Very serious	655 (67.9)
Somewhat serious	188 (20.9)
Not serious	90 (9.5)
Not very serious	16 (1.7)
**How serious is the problem TB in your working area?**	
Very serious	294 (30.2)
Somewhat serious	223 (22.9)
Not serious	249 (25.6)
Not very serious	207 (21.3)
**What would be your reaction if you were found to have TB?**	
Fear	379 (39.9)
Surprised	80 (8.4)
Shame	14 (1.5)
Sadness or hopeless	123 (13.0)
No special feeling	353 (37.2)
**Do you think some people are more likely to become infected than others?**	
Yes	268 (28.2)
No	681 (71.8)
**How is your feeling towards people with TB?**	
Compassion and desire to help	583 (61.4)
Compassion but stay away from them	109 (11.5)
It is their problem and I cannot get TB	9 (1.0)
I fear them because they may infect me	94 (9.9)
I have no particular feeling	154 (16.2)
**In your community (working area) how is a person who has TB usually regarded/treated?**	
Most people reject him or her	81 (8.5)
Most people are friendly, but they try to avoid him/ her	398 (42.1)
Mostly supports and helps him/ her	299 (31.5)
not sure whether they help/ not	97 (10.2)
Don’t give special attention	74 (7.7)
**The overall favorable attitude**	
Poor attitude	470 (49.5)
Favorable attitude	479 (50.5)

#### Health seeking behavior

Four hundred ninety-two (51.82%) respondents would go to doctor/health professional if they had TB. Besides, 175 (18.44%) and 175 (18.44%) respondents respond they would contact parents and close friends, respectively if they had TB. Of all, 708 (74.60%) would seek medical care if the symptoms last for greater than 2 weeks. Two hundred (21.07%) would seek medical care as soon as they realized the symptoms. The remaining, 41 (4.32%) would seek medical care when my treatment doesn’t work. From 552 respondents with poor knowledge, 62 (11.23%) had good seeking behavior. On the other hand among 397 respondents with good knowledge 93 (23.43%) had good health-seeking behavior.

#### Associated factors with knowledge of respondents

In the bivariate analysis seven factors including educational status, family size, daily income, mass media exposure, obtain health information from friends, health information from posters, and hearing health information from school became significant at *p*-value less than 0.2. However, in multivariable analysis, only family size and mass media exposure retained significantly. The odds of having good knowledge among respondents having 3–5 and > =6 families were increased by 80 and 85%, respectively compared to respondents having less than three families. The odds of good knowledge were increased by 42% among respondents having mass media exposure compared to their counterparts (Table 4).

**Table 4:** Bi-variable and multivariable logistic regression analysis among migrants and seasonal farmworkers on the of knowledge about tuberculosis in West Armacho, Ethiopia (*N* = 949)

Characteristics	Knowledge status		COR with 95% CI	AOR with 95%
	Good	Poor		
**Education status**				
Unable to read and write	147	227	1	1
Able to read and write	29	44	1.02 (0.61–1.70)	0.94 (0.56, 1.59)
Primary	180	225	1.24 (0.93, 1.64)	1.09 (0.81, 1.49)
Secondary and above	41	56	1.13 (0.72, 1.78)	0.87 (0.52, 1.45)
**Family size**				
1–2	28	60	1	1
3–5	227	309	1.57 (0.97, 2.54)	1.80 (1.09,2.96)^*^
> =6	142	183	1.66 (1.01, 2.74)	1.85 (1.11,3.10)^*^
**Daily income**				
Low	222	274	1.29 (0.99, 1.67)	1.28 (0.98,1.67)
High	175	278	1	1
**Mass media exposure**				
No	228	393	1	1
Yes	169	159	1.83 (1.39, 2.40)	1.42 (1.02,2.01)^*^
**Friends as sources of information**				
No	252	427	1	1
Yes	145	125	1.97 (1.48,2.61)	1.41 (0.97,2.05)
**Poster as sources of information**				
No	342	517	1	1
Yes	55	35	2.38 (1.51,3.71)	1.65 (0.97, 2.83)
**Scholars sources of information**				
No	321	483	1	1
Yes	76	69	1.66 (1.16, 2.36)	1.07 (0.68,1.70)

*p-value < 0.05

#### Associated factors with attitude of respondents

In the bivariate analysis educational status, occupation, family size, residency, health information, and knowledge were statistically significant. However, in the multivariate logistic regression analysis, only three variables (Health information, residence, and knowledge) were statistically significant factors to have a favorable attitude. The odds of having a favorable attitude among urban dwellers were increased by 63% compared with rural counterparts. The odds of favorable attitude on TB among respondents who had health information were increased by 72% compared with their counterparts. The odds of favorable attitudes were 4.3 times higher among participants with good knowledge compared with their counterparts (Table 5).

**Table 5:** Factors related to the level of attitude about tuberculosis among West Gondar, Ethiopia Migrants and seasonal workers in the bivariate and multivariate logistic regression analysis (*N* = 949)

Variables	Attitude status		COR with 95% CI	AOR with 95% CI
	Favorable	unfavorable		
**Educational status**				
Unable to read and write	180	194	1	1
Able to read and write	30	43	0.75 (0.45,1.25)	0.68 (0.39, 1.17)
Primary	208	197	1.14 (0.86,1.5)	0.68 (0.64, 1.25)
Secondary and above	61	36	1.83 (1.15,2.89)	1.31(0.75,2.29)
**Residence**				
Rural	407	424	1	1
Urban	72	46	1.63(1.09,2.42)	1.66 (1.05,2.62)^*^
**Occupation**				
Student	108	69	1	1
Farmer	309	335	0.59 (0.42,0.83)	0.72 (0.47,1.10)
Unemployed	62	66	0.60 (0.38,0.95)	0.68 (0.40,1.15)
**Family size**				
1–2	37	51	1	1
3–5	278	258	1.49 (0.94,2.34)	1.50 (0.90,2.49)
6 and above	161	164	1.40 (0.87,2.26)	1.37 (0.81,2.34)
**Health information**				
No	163	221	1	1
Yes	316	249	1.72 (1.32,2.24)	1.64 (1.23,2.18)^*^
**Knowledge status**				
Poor knowledge	198	354	1	1
Good knowledge	281	116	4.33 (3.28,5.72)	4.33 (3.26, 5.75)^*^

*p-value < 0.05

## Discussion

This study revealed that there are a low knowledge and attitude on TB among migrant and seasonal farmworkers. Only 41.83% of migrants and seasonal farmworkers were knowledgeable. Furthermore, 50.47% had a favorable attitude towards TB. TB knowledge among migrants and seasonal farmworkers were significantly affected by mass media exposure and family size. On the other hand, their level of attitude was affected by health information, residence, and knowledge of respondents. Besides, 708 (74.60%) would seek medical care if the symptoms last for greater than 2 weeks.

In our study, 41.85% of migrant and seasonal farmworkers were knowledgeable. This finding was in line with the finding from EDHS 2011 (44.14%) [17]. However, the finding was lower than a study conducted in Lesotho (59.9%) (22)and Zimbabwe (73.8%) [22]. This could be due to almost all (98.3%) of respondents in Lesotho and 93% in Zimbabwe had formal education. However, only 52.90% of respondents had formal education in the current study. This could be because a high level of education is usually catalyzed awareness of TB, which acts as a precursor to having good knowledge [23]. Also, a study from Indonesia showed that education is an antecedent of knowledge and has a great contribution to fortifying respondent’s knowledge [24]. A case-control study conducted in Sudan showed that 66.5% of respondents had good knowledge which is higher than the present study [19]. This could be due to nearly half of the respondents were cases, which have information about their disease status and get advice during their follow up. Furthermore, a study from Iran showed that 62.04% had good knowledge [25]. This difference could be due to defining the outcome variable. In the current study, good knowledge was defined if a respondent correctly answered more than half of the questions. Whereas, in Iran, study mean score was used to classify as knowledgeable.

There is a significant increase in knowledge among mass media exposed migrants and seasonal farmworkers. This finding is in agreement with a study conducted in a general population of Lesotho [26] and India [27]. This is because mass media campaign has a big role in enhancing the normal passive case finding strategy by reaching a large population at a time and it provides information regarding on earliest symptoms, cause, and transmission, prevention, and treatment modalities. Accordingly, a study from Colombia showed that mass media exposure was associated with a high level of knowledge and recommended that it should be sustainable to have a long term change to adopt behavioral change and create a habit to use [28]. Moreover, health information through mass media and other methods was significantly affecting the attitude of respondents. This could be the fact that mass media has a great role in creating awareness [29], which can later change perceptions and behaviors’ of respondents [30, 31].

Family size is another significant factor affecting the knowledge status of migrants and seasonal farmworkers. This finding is supported by a study conducted in Thailand among the general population and risk groups [18]. This could be due to the culture of sharing information among the family members to one another if family size is large. Also, in a large family, there could be at least one educated person who could transmit a message regarding TB during any discussion in the household.

Nearly half of migrants and seasonal farmworkers had a favorable attitude. This finding is lower than a study conducted in Timor Leste (83.3% in men and 88.6% in women) [32]. This could be due to the difference in defining the outcome variable. In the former study, a favorable attitude was measured for specific questions (intention to initiate TB treatment). However, in the current study favorable attitude was measured by creating a composite score and the one who scored more than half the attitude question. Similarly, our study was much lower than a community study from Botswana which showed about 92% of respondents to have a favorable attitude [33].

Good knowledge was associated with a favorable attitude. This finding is supported by a study conducted in Nigeria [7]. This could be because of good knowledge influence attitude formation [30]. Furthermore, it was supported by a study from Ethiopia among pastoralist communities which showed lower awareness as risk factors for unfavorable attitudes [34]. This could be mainly due to perceiving a higher stigma on TB patients among pastoralists with poor awareness.

The strength of the study was determining knowledge, attitude, and factors on TB among migrant and seasonal farmworkers in Ethiopia. This is the first study to evaluate migrant’s knowledge and attitude in the context of one of the developing countries, Ethiopia. This study has some limitations. Initially, the study could not allow establishing cause-effect relationships because of cross-sectional nature. Besides, this study could be vulnerable to social desirability bias. There was also a scarcity of literature among migrants and seasonal farmworkers in developing countries. The conducted studies have relied on the general population not specifically on migrants. Finally, this study also suffers from recall bias due to data was collected about their previous experience.

The findings of this study would contribute significantly to design tailored interventions intended in increasing awareness through the use of mass media and developing a favorable attitude in migrants and seasonal farmworkers towards TB. Furthermore, this finding provokes policymakers to design programs and to implement appropriate public health strategies targeted migrants and seasonal farmworkers. As a result, improving the level of knowledge and attitude has paramount importance in the control and elimination of TB cases. Therefore, it is compulsory to do on migrants and seasonal farmworkers to control and eliminate TB in the country.

## Conclusion

This study revealed low overall knowledge and attitudes of migrant and seasonal farmworkers. Mass media exposure and family size significantly affect the knowledge of respondents. Moreover, health information, urban residency, and good knowledge were the main identified factors of a favorable attitude. Health education interventions, focused on TB cause, mode of transmission, prevention, and treatment are important to migrants and seasonal farmworkers. Besides, the role of mass media should be strengthened to improve the knowledge and attitude of migrants and seasonal farmworkers.

## Supplementary information

**Additional file 1.** The questionnaire developed for this study is provided as Additional File 1.

## Data Availability

Data will be available from the corresponding author upon request.

## Abbreviations

AOR: Adjusted Odds Ratio
BSc: Bachelor of Science
CI: Confidence Interval
COR: Crude Odds Ratio
EDHS: Ethiopia Demographic Health Survey
HIV: Human Immunodeficiency Virus
IQR: Inter Quartile Range
MDR: Multi-Drug Resistance
TB: Tuberculosis
WHO: World Health Organization
