# Supplementary material for: The role of mass media exposure on tuberculosis knowledge and attitude among migrant and seasonal farmworkers in Northwest Ethiopia
Source: BMC Infect Dis. 2020 Aug 5;20:579. doi: 10.1186/s12879-020-05316-9 (PMC7405343; doi:10.1186/s12879-020-05316-9)
Supplement: Supplementary file 1 — Additional file 1. The questionnaire developed for this study is provided as Additional File 1. [file 12879_2020_5316_MOESM1_ESM.docx]

**Consent and Information sheet**

**Title of the research project:** Health issues of migrant and seasonal farm workers in Amhara Regional state, northwest Ethiopia

**Principal Investigator**: Getu Debalkie

**Name of the organization**: University of Gondar, College of Medicine and Health Sciences, Institute of Public Health.

**Introduction**

My name is _____________ and I am a data collector. Right now I am going to give you the

relevant information concerning the research and I invite you to be part of this research. Before you decide to be part of the research you can talk to anyone to feel comfortable with the research. If there is any word that you do not understand while I am giving the information, please stop me and ask me and I will explain to you. The aim of the project is to identify the health issues of migrant and seasonal farm workers and multidimensional impact of mobility on themselves, families and community in Amhara Regional State, northwest Ethiopia. There is no risk or direct benefit in participating in this research project. You will not be provided any incentives or payment to take part in this project. Participating and not participation is the full right of participants and they can stop participating in the study at any time. They can also skip any question which they do not want to respond. They can ask any question which is not clear for them. It will take 30 minutes. Are you voluntary to

| participate? Yes | | | | No (Stop) | |  |  |  |  |  |
| --- | --- | --- | --- | --- | --- | --- | --- | --- | --- | --- |
| **Person to contact** | | | | | |  |  |  |  |  |
|  | 1. | | | Getu Debalkie (Principal investigator, University of Gondar), phone: +251 916747030; | | | | | | |
| E-mail: [getud2006@gmail.com](mailto:getud2006@gmail.com) | | | | | |  |  |  |  |  |
|  |  |  |  |  |  | Code_____________________________ | | | | |
| **Permanent residence** | | | | | |  |  |  |  |  |
| Regional State: __________________Zone:________________District: _______________ | | | | | | | | | | |
| **Farming area** | | | |  |  |  |  |  |  |  |
| District: __________________Company name:________________ | | | | | | | |  |  |  |
| **Part I: Socio demographic characteristics** | | | | | |  |  |  |  |  |
|  |  |  |  | | |  |  | |  |  |
| S.No |  |  | Questions | | |  | Response | | |  |
| 101. |  |  | Sex | | |  | 1. Male | 2. Female | | |
|  |  |  | | | |  | |  |  |  |
| 102. |  | Age (in years) | | | | ------------- | |  |  |  |
| 103. What is the highest education level you | | | | | | 3. | 1-4 Grades |  | 6. | 11-12 Grades |
|  |  | completed? | | | | 4. | 5-8 Grades |  | 7. | Diploma/above |
|  |  | 1. Unable to read and write | | | | 5. | 9-10 Grades |  |  |  |
|  |  | 2. Able to read and write without formal | | | |  |  |  |  |  |
|  |  |  | Education | | |  |  |  |  |  |

1. Main occupation before departure

|  | 1. | Student | 2. Farmers | 3. Housemaid | |
| --- | --- | --- | --- | --- | --- |
|  | 4 | Merchant | 5. Unemployed |  | 6. Others (specify) ______ |
|  |  | |  |  |  |
| 105. | Family size (in number) | |  |  | ______ |
| 106. | Residence | | 1. Rural | 2. Urban | |

1

|  | 107. | |  | |  | Ethnicity 1.Amhara 2. Kimant 3. Tigre 4. Others (Specify) | | | | | | | | | | | | | | | | | | | | ___ | | | |  | | | | |  | | | |  |  |
| --- | --- | --- | --- | --- | --- | --- | --- | --- | --- | --- | --- | --- | --- | --- | --- | --- | --- | --- | --- | --- | --- | --- | --- | --- | --- | --- | --- | --- | --- | --- | --- | --- | --- | --- | --- | --- | --- | --- | --- | --- |
|  | 108. | |  | |  | What is your religion? 1. Orthodox | | | | | | | 2. Muslim | | | | | | 3. Protestant | | | | | | | | | | | 4. Catholic | | | | | 5. Other | | | |  |  |
|  |  | |  | |  | (specify)_____________ | | | |  | | |  | | |  |  | |  | |  |  |  | |  |  | | | |  | | | | |  | | | |  |  |
|  |  | |  | |  |  | | | |  | | |  | | |  |  | |  | |  |  | | | | | | | |  | | | | |  | | | |  |  |
|  | 109. | |  | |  | Marital status 1. Single | | | | 2. Married | | | 3. Divorced | | | | | |  | | 4. Widowed | | | | | | | | | 5. Separated | | | | | | | | |  |  |
|  |  | |  | |  |  | |  |  |  | | |  | | |  |  | |  | |  |  | | | | | | | | | | | | |  | | | |  |  |
|  | 110. | |  | |  | Salary | |  |  |  | | |  | | |  |  | | _________ETB(**Daily**) | | | | | | | | | | | | | | | |  | | | |  |  |
|  | 111. | |  | |  | How many times have you been here? | | | | | | | | | |  |  | | ______times | | | | | | | | | | |  | | | | |  | | | |  |  |
|  | 112. | |  | |  | How long have you been living here in the | | | | | | | | | |  |  | | _____Days | | | | | | | | | | |  | | | | |  | | | |  |  |
|  |  | |  | |  | current visit? | | |  |  | | |  | | |  |  | |  | |  |  |  | |  |  | | | |  | | | | |  | | | |  |  |
| **Part II. Source of information about health** | | | | | | | | | | | | |  | | |  |  | |  | |  |  |  | |  |  | | | |  | | | | |  | | | |  |  |
|  |  | |  | |  |  | | |  |  | | |  | | |  |  | |  | |  |  |  | |  |  | | | | | | | | |  | | | |  |  |
|  | S.No | |  | |  | Questions | | |  |  | | |  | | |  |  | |  | |  |  |  | |  | Response | | | | | | | | | Remark | | | |  |  |
|  | 201. | | Have you seen or heard any health information | | | | | | | | | | | | | | | | | |  |  |  | | 1. | Yes | | | | 2. No | | | | |  | | | |  |  |
|  |  | |  | | messages from any source recently? | | | | | | | |  | | |  |  | |  | |  |  |  | |  |  | | | |  | | | | |  | | | |  |  |
|  |  | |  | | | | | | | | | | | | | | | |  | |  |  |  | |  |  | | | |  | | | | |  | | | |  |  |
|  | 202. | | What was the source of information about health? | | | | | | | | | | | | | | | |  | |  |  |  | |  |  | | | |  | | | | |  | | | |  |  |
|  |  | |  | |  | 202.1. Mass media (Radio/ TV) | | | | | | |  | | |  |  | |  | |  |  |  | | 1. | Yes | | | | 2. No | | | | |  | | | |  |  |
|  |  | |  | |  | 202.2. Health workers | | | | | | |  | | |  |  | |  | |  |  |  | | 1. | Yes | | | | 2. No | | | | |  | | | |  |  |
|  |  | |  | |  | 202.3. Friends/Family | | | | | | |  | | |  |  | |  | |  |  |  | | 1. | Yes | | | | 2. No | | | | |  | | | |  |  |
|  |  | |  | |  | 202.4. Posters/notices/magazines/newspaper | | | | | | | | | | | | | | |  |  |  | | 1. | Yes | | | | 2. No | | | | |  | | | |  |  |
|  |  | |  | |  |  | | | |  | | |  | | |  |  | |  | |  |  |  | |  |  | | | |  | | | | |  | | | |  |  |
|  |  | |  | |  | 202.5. School | | | |  | | |  | | |  |  | |  | |  |  |  | | 1. | Yes | | | | 2. No | | | | |  | | | |  |  |
|  |  | |  | |  |  | | | | | | |  | | |  |  | |  | |  | | | | | | | | | | | | | |  | | | |  |  |
|  |  | |  | |  | 202.6. Others(specify) | | | | | | |  | | |  |  | |  | | _____________________ | | | | | | | | | | | | | |  | | | |  |  |
| **Part III. Knowledge attitude and practice towards TB** | | | | | | | | | | | | | | | | | | | | |  | | |  | | |  |  |  | |  |  |  |  | |  |  |  |  |  |
|  | | | |  | | | | | | |  |  | |  |  | | |  | |  |  | | | | | | | | | |  |  |  |  | |  |  |  |  |  |
| **S.No** | |  | | **Knowledge** | | | | | | |  |  | |  |  | | |  | |  | **Response** | | | | | | | | | |  |  |  |  | |  |  |  |  |  |
| 801 | |  | | Is the cause of TB bacteria or germ | | | | | | |  |  | |  |  | | |  | |  | 1. | | | | | | Yes | | | | 2. No | | | | |  |  |  |  |  |
|  | |  | |  | | | | | | |  |  | |  |  | | |  | |  |  | | |  | | |  |  |  | |  |  |  |  | |  |  |  |  |  |
| 802 | |  | | Sign and symptoms of TB | | | | | | |  |  | |  |  | | |  | |  |  | | |  | | |  |  |  | |  |  |  |  | |  |  |  |  |  |
|  | |  | |  | | | | | | | | | |  | | | | | |  |  | | |  | | | | | | | | | | | |  | |  |  | |
|  | |  | | 1. Persistent cough ≥ 2 weeks 1.Yes | | | | | | | | | | 2. No | | | | | | |  | | | 4. Night sweating | | | | | | | | | | | | 1.Yes | |  | 2. No | |
|  | |  | | 2. | | | Sputum with blood | | | | 1.Yes |  | | 2.No | | | | | | |  | | | 5. Chest pain | | | | | | | | | | | | 1.Yes | |  | 2. No | |
|  | |  | | 3. Fever | | | | | | | 1.Yes | | | 2.No | | | | | | |  | | | 6. Weight loss | | | | | | | | | | | | 1.Yes | |  |  | 2.No |
|  | |  | | 4. poor appetite | | | | | | | 1.Yes | | | 2.No | | | | | | |  | | |  | | |  |  |  | |  |  |  |  | |  |  |  |  |  |
| 803 | |  | | Can TB be transmitted | | | | | | |  |  | |  |  | | |  | |  | 1. | | | | | | Yes | | | |  |  | 2.No | | |  |  |  |  |  |
|  | |  | |  | | | | | | |  |  | |  |  | | |  | |  |  | | |  | | |  |  |  | |  |  |  |  | |  |  |  |  |  |
| 804 | |  | | TB mode of transmission | | | | | | |  |  | |  |  | | |  | |  |  | | |  | | |  |  |  | |  |  |  |  | |  |  |  |  |  |
|  | |  | | 1. | | | Air when coughing or sneezing | | | | | 1.Yes | |  | 2.No | | | | | | 4. Sexual contact | | | | | | | | | | | | | | | 1.Yes | | 2.No | | |
|  | |  | | 2. | | | Touching a person with TB | | | | | 1.Yes | | | 2.No | | | | | | 5. Drinking unboiled milk | | | | | | | | | | | | | | | 1.Yes | | 2.No | | |
|  | |  | | 3. | | | Sharing utensils | | | |  | 1.Yes | | | 2.No | | | | | | 6. Other__ | | | | | | | | | |  |  |  |  | | 1.Yes | | 2.No | | |
|  | |  | |  | | | | | | |  |  | |  |  | | |  | |  |  | | | | | |  | | | |  | | | | |  |  |  |  |  |
| 805 | |  | | Can TB be prevented | | | | | | |  |  | |  |  | | |  | |  | 1. | | | | | | Yes | | | | 2. No | | | | |  |  |  |  |  |
| 806 | |  | | Preventive method of TB | | | | | | |  |  | |  |  | | |  | |  |  | | |  | | |  |  |  | |  |  |  |  | |  |  |  |  |  |
|  | |  | | 1. | | | Minimizing close contact | | | | 1.Yes | | |  | 2. No | | | | | |  | | | 5. Good nutrition | | | | | | | | | | | | 1.Yes | |  |  | 2. No |
|  | |  | | 2. | | | Covering mouth while coughing 1.Yes | | | | | | |  | 2. No | | | | | |  | | | 6. Using separate rooms | | | | | | | | | | | | 1.Yes | |  |  | 2. No |

12

|  | 3. | Avoid sharing utensils | 1.Yes | 2. No | 7. Closing opening window1.Yes | | 2. No |
| --- | --- | --- | --- | --- | --- | --- | --- |
|  | 4. | Earlytreatment | 1.Yes | 2. No | 8. Other_ |  |  |
| 807 | TB is treatable | |  |  | 1. Yes | 2. No |  |
|  |  | |  |  |  |  |  |
| 808 | Treatment method of TB 1. Modern drug | | | 2. Nutritional support | | 3. Other_____ |  |

| **B.Attitude** | | | | | | | | | | |
| --- | --- | --- | --- | --- | --- | --- | --- | --- | --- | --- |
| 809 | How serious is the diseases TB | | | | |  |  |  |  |  |
|  | 1. | Very serious | | 2. Somewhat serious | | | 3. Not serious | | 4. Not very serious |  |
| 810 | How serious the problem TB in your working area | | | | | | | |  |  |
|  | 1. | Very serious | | 2. Somewhat serious | | | 3. Not serious | | 4. Not very serious |  |
| 811 | What would be your reaction if you were found that you have TB | | | | | | | | |  |
|  |  |  |  | | |  |  |  |  | |
|  | 1. | Fear | 2. surprised | | | | 3. Shame | | 4. Sadness or hopeless 5. Other____ | |
| 812 | Do you think some people are more likely to | | | | | | become infected than others? 1. Yes | | | 2. No |
| 813 | If yes for the above question who is more likely to be infected | | | | | | | |  |  |
|  | 1. | Women | 2. Men | | 3. Both men and women 4. Older people 5. Children<5 6. Other____ | | | | | |
| 814 | Do you know people with TB? 1. Yes 2. No | | | | | | | |  |  |
|  | How is your feeling toward people with TB | | | | | |  |  |  |  |
| 815 | **1**. Compassion and desire to help | | | | | **2**. Compassion but stay away from them **3**.It is their problem and I | | | | |
|  | cannot get TB **4**. I fear them because they may infect me **5**. I have no particular feeling. | | | | | | | | | |
| 816 | In your community(working area) how is a person who has TB usually regarded/treated | | | | | | | | | |
|  | **1**. Most people reject him or her **2**. Most people are friendly, but they generally try to avoid him or her | | | | | | | | | |
|  | **3**. Mostly supports and helps him or her **4**. not sure whether they help or not, **5**. Don’t give special | | | | | | | | | |
|  | attention, 6. other______________ | | | | | |  |  |  |  |
| **C. Seeking behavior** | | | | | | | | | | |
| 817 | Who would you talk to about your illness if you had TB | | | | | | | |  |  |
|  | **1**. Doctor or other medical worker **2**. Spouse **3**.Parent **4**. Close friend **5**. No one 6.Other_____ | | | | | | | | | |
|  |  | | | | | | | | |  |
| 818 | If you had symptoms of TB, at what point would you seek medical help | | | | | | | | |  |
|  | **1**. When treatment on my own does not work **2**. When TB symptoms last for 2 or more weeks **3**. As soon | | | | | | | | | |
|  | as I realize TB symptoms | | | | **4**. I would go to health facility or contact a doctor **5**.Other________ | | | | | |
| 819 | If you would not go to the health facility, what is the reason | | | | | | | |  |  |
|  | 1. Not sure where to go **2**. Cost | | | | | **3**. Difficulties with transportation/distance **4**. Do not trust medical | | | | |
|  | workers **5**. Do not like attitude of medical workers **6**. Can not leave my work | | | | | | | | | **7**. Do not want to find |
|  | that something is wrong **8**. Other_________________________ | | | | | | | | |  |

13
